# Supplementary material for: TIM3 Expression in Anaplastic-Thyroid-Cancer-Infiltrating Macrophages: An Emerging Immunotherapeutic Target
Source: Biology (Basel). 2022 Nov 3;11(11):1609. doi: 10.3390/biology11111609 (PMC9687546; doi:10.3390/biology11111609)

Supplementary Figures

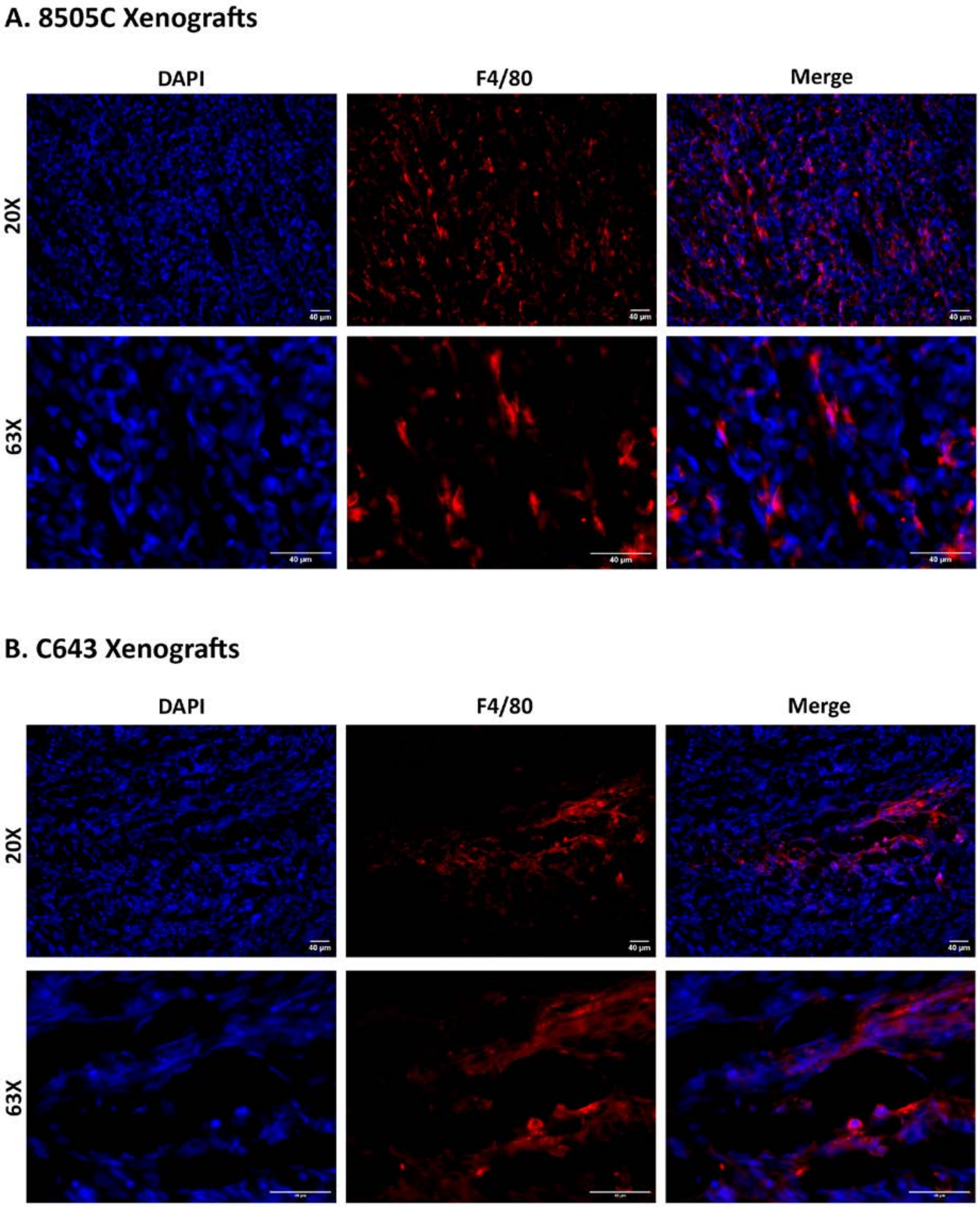

Supplementary Figure S1. Macrophages are recruited to ATC xenograft tumors. 20X (top) and 63X (Bottom) images of 5 μm thick 8505C (A) and C643 (B) xenograft tumors section immunostained with F4/80-PE antibody (red) to confirm presence of macrophages. Nuclei are counterstained with DAPI (blue).

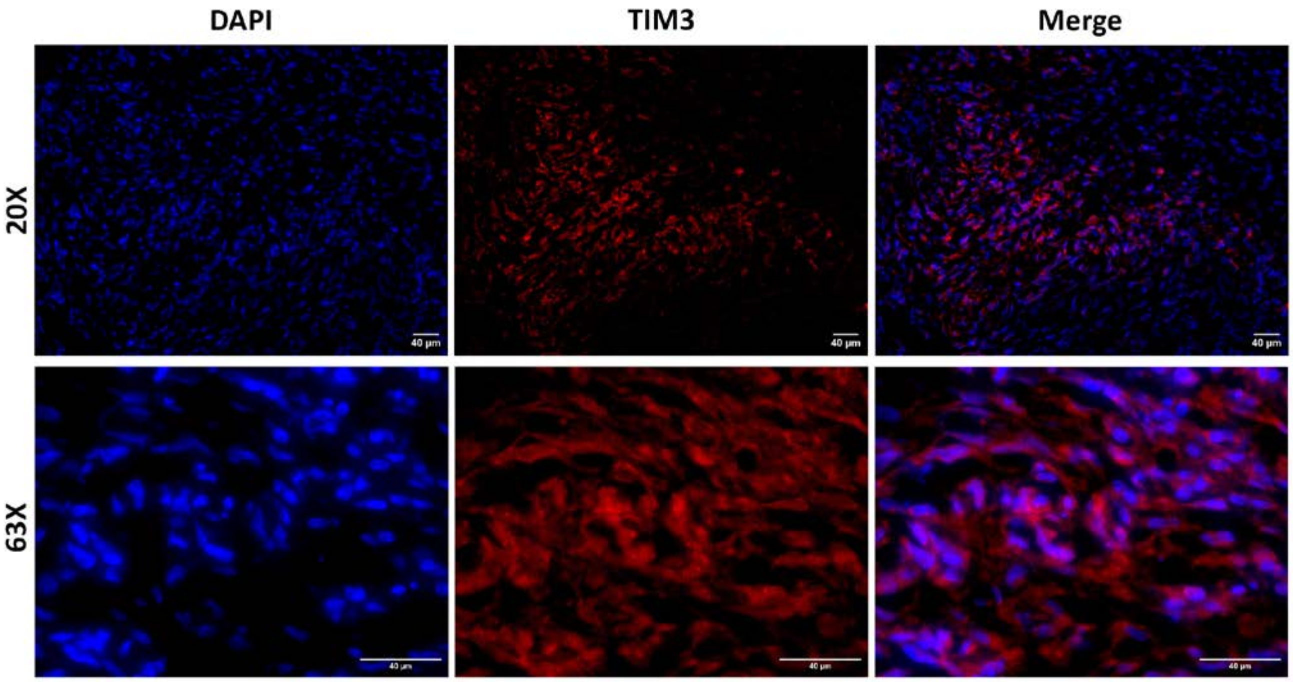

Supplementary Figure S2. TIM3 staining was observed within C643 xenograft tumors. 20X (top) and 63X (Bottom) images of 5 μm thick C643 xenograft tumors section immunostained with TIM3-PE antibody (red). Nuclei are counterstained with DAPI (blue).

This figure displays the full-length gels for Figure 4 panel A-I shown in the text/Results.

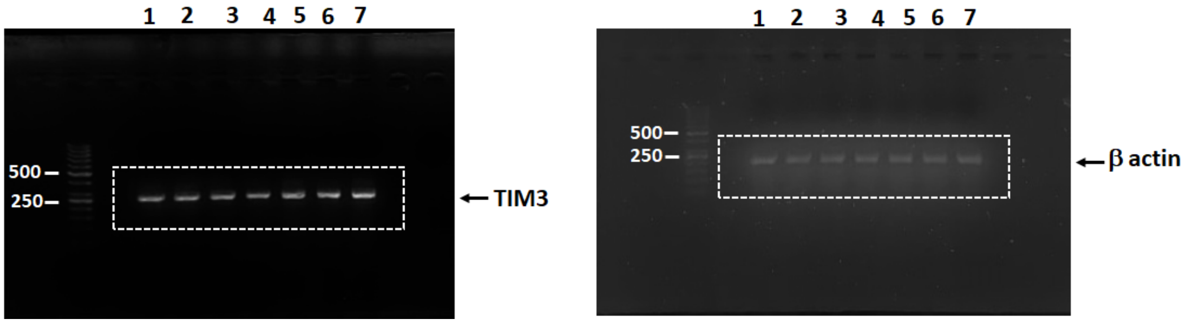

Supplement: Supplementary file 1 [file biology-11-01609-s001.zip › biology-1922530-supplementary.pdf]
